# Bibliography

**Published:** 1903-11-15

**Authors:** 


### BIBLIOGRAPHY.

The well-known book publishing house of Lea Brothers & Co. are bringing out a series of epitomized medical science books under the supervision of Victor C. Petersen, A.M., M.D. It is the purpose to have each volume contain a concise statement of present-day knowledge of each subject treated. The books are so arranged that they may be advantageously used by students as well a practitioners in reviewing any subject. At the end of each chapter is printed a list of questions calculated to bring out, by self-examination, the facts treated in the chapter. In this feature they differ from ordinary quiz compends as no answers to the questions are given, the student must consult the text for answers, and he will be greatly assisted here as the text is given in consecutive but remarkably clear language. Two volumes are at hand, one on Microscopy and Bacteriology, and the other on Physics and Inorganic Chemistry.

The manual on "*Microscopy and Bacteriology*" is compiled by P. E. Archinard, A.M., M.D., of New Orleans and covers the subject of Bacteriology in its essentials in a very satisfactory manner. The biological and technical as well as the therapeutic aspect of the subjects is concisely stated. Illustrations are used where necessary and several colored plates are used to good advantage to save long descriptions and to elucidate the subject.

The manual on "*Physics and Inorganic Chemistry*" is compiled by A. McGlannon, M.D., of Baltimore. Part 1 of this manual is given up to Physics and treats of the general definitions and the fundamentals of heat, light, electricity and magnetism. Part 2 gives a brief but satisfactory statement of the fundamental principles of general chemistry and then takes up the important inorganic elements singly and in their normal groups. The author has drawn largely from the work of Prof. Simons, assuring accuracy as well as advanced scientific statements.

The books are well made and would be valuable addition to any library, as they will answer quickly many questions more satisfactorily than the dictionaries, and will also serve well for review purposes.

